# Bone health in avoidant/restrictive food intake disorder: a narrative review

**DOI:** 10.1186/s40337-023-00766-3

**Published:** 2023-03-22

**Authors:** Kaitlin B. Proctor, Eugene Rodrick, Staci Belcher, William G. Sharp, Joseph M. Kindler

**Affiliations:** 1 Emory University School of Medicine and Children’s Healthcare of Atlanta, Athens, GA USA; 2 Department of Nutritional Sciences, University of Georgia, Room 279 Dawson Hall, 305 Sanford Drive, Athens, GA 30606 USA

**Keywords:** Avoidant/restrictive food intake disorder, Bone mineral density, Bone accrual, Anorexia nervosa

## Abstract

**Background:**

Avoidant/restrictive food intake disorder (ARFID) is an eating/feeding disturbance characterized by severe food avoidance or restriction that results in faltering growth, nutritional deficiencies, dependence on formula supplementation, and/or significant psychosocial impairment. Compared to other eating disorders, ARFID is observed to have an earlier childhood onset and chronic course without intervention. Childhood represents a sensitive period for longitudinal growth and bone accrual, setting the stage for long-term health outcomes associated with longevity and quality of life, including risk for fracture and osteoporosis.

**Results:**

This narrative review discusses published scientific literature on bone health in individuals with ARFID by describing the current understanding of ARFID’s effect on bone health, how common dietary constraints characteristic of ARFID may present unique risks to bone health, and the current clinical recommendations for bone health assessment. Reviewing what is known of clinical data from anorexia nervosa (AN) and similar cohorts, the chronicity and etiology of dietary restriction observed in ARFID are hypothesized to compromise bone health significantly. Although limited, examination of bone health in ARFID patients suggests children with ARFID tend to have shorter stature compared to healthy reference datasets and have lower bone density compared to healthy individuals, similar to those with AN. There remains a substantial knowledge gap in how ARFID may interrupt bone accrual during childhood and adolescence, and subsequent impact on attainment of peak bone mass and peak bone strength. The longitudinal effects of ARFID may be subtle and overlooked clinically in the absence of severe weight loss or growth stunting. Early identification and remediation of threats to bone mass accrual have significant personal and population-level implications.

**Conclusion:**

For patients with ARFID, delayed identification and intervention to address feeding disturbances may have a long-lasting impact on various body systems and processes, including those relating to longitudinal growth and bone mass accrual. Further research employing rigorous prospective observational and/or randomized study designs are required to clearly define effects of ARFID, as well as clinical interventions aimed at addressing ARFID-related feeding disturbances, on bone accrual.

## Abstract (plain language)

An extensive body of evidence reports threatened bone health in people with eating disorders, focusing primarily on people with anorexia nervosa. The Diagnostic and Statistical Manual of Mental Disorders (DSM) Fifth Edition reclassified and expanded upon the DSM-IV diagnosis of “feeding disorder of infancy or early childhood,” introducing a new diagnostic term, avoidant/restrictive food intake disorder (ARFID). This has led to an increased need to understand effects of ARFID on bone health. Among the studies that have reported bone health outcomes in people with ARFID, authors have reported shorter stature and lower bone mineral density (BMD) in children and adults with ARFID compared to reference datasets. Malnutrition resulting in under-consumption of energy and/or nutrients that are integral to bone health can also cause conditions that impact the musculoskeletal system, including low body weight, scurvy due to vitamin C deficiency, and rickets due to vitamin D deficiency. Research in individuals with ARFID focusing on longitudinal changes in BMD, bone micro-structure, and bone strength during clinical intervention are required. These efforts will help identify long-term health risks in people with ARFID, inform comprehensive medical assessment, improve long-term health outcomes, and provide a benchmark for assessing treatment outcomes over time.

## Background

In 2013, the Diagnostic and Statistical Manual of Mental Disorders Fifth Edition (DSM-5) reclassified and expanded upon the DSM-IV diagnosis of “feeding disorder of infancy or early childhood,” introducing a new diagnostic term, avoidant/restrictive food intake disorder (ARFID) [1]. ARFID was placed within an expanded “feeding and eating disorder” section alongside pica, anorexia nervosa (AN), bulimia nervosa (BN), binge-eating, and rumination disorder. ARFID’s clinical heterogeneity manifests in four subtypes: faltering growth (A1 subtype), nutritional deficiencies (A2 subtype), reliance on enteral or oral formula supplementation (A3 subtype), and/or marked interference with psychosocial functioning (A4 subtype) [1]. A patient may present with multiple clinical manifestations and a thorough understanding of these ARFID subtypes is crucial, as subtype diagnosis guides how assessment and treatment targets are prioritized. The treatment of ARFID broadly aims to ensure inclusion of sufficient food volume and variety to improve patient health and their relationship to food and mealtimes, varying across individual patients based on unique needs. As an example, treatment for individuals who are underweight or formula dependent begins with a focus on increasing overall volume and variety of foods consumed, whereas individuals with nutritional deficiencies need only to focus on a variety of foods consumed.

ARFID may also co-occur with a variety of medical conditions that are attributed to and/or perpetuated by consumption of specific nutrients and/or foods, as in celiac disease, lactose intolerance, food allergy, among many others. However, an ARFID diagnosis is only appropriate if the restrictive/selective eating behaviors extend beyond the specific dietary restrictions as dictated by medical needs. As an example, an individual with food allergies to wheat and dairy would not necessarily meet criteria for ARFID because foods that contain these allergens are strictly excluded from the diet. For consideration of an ARFID diagnosis, there would need to be food restrictions that extend beyond the medically required restriction and/or if the individual experiences significant impairment in their ability to eat outside of the home or in social settings. In pediatrics, medical or developmental conditions increase the risk for feeding disorder significantly, with 40–80% of these children meeting criteria for a feeding disorder. Thus, thorough assessment of comorbid medical conditions, particularly those that cause pain or discomfort with eating, is crucial to ensure appropriate diagnosis and treatment [2–6].

It is generally understood that ARFID may portend numerous health consequences secondary to malnutrition, as observed in other eating disorders such as AN [7]. The symptom onset of ARFID is typically reported in early childhood and persists over time [8]. It is hypothesized to be developed and maintained by a complex set of neurobiological and psychological factors including low appetite or interest in eating, heightened sensory sensitivity to food characteristics, and/or fear of adverse consequences due to eating [3]. While patients in the A1 subtype consistently present with significant weight loss or chronic failure to gain weight due to restricted food intake, weight loss/faltering growth is not required for an ARFID diagnosis. ARFID differs from other eating disorders involving dietary restriction (e.g., AN and BN) in that the severely restricted food intake is not attributed to disrupted body image or efforts to control weight. As such, patients with ARFID may have weight loss/faltering growth, but many present without frank growth impairment. Additionally, patients with ARFID likely have different macronutrient and micronutrient consumption profiles compared to other eating disorders. For example, patients who present with severe food selectivity often consume diets high in processed foods, carbohydrates, and added sugars, with low intake of fruits, vegetables, and proteins [9]. These diets place patients at risk of consistently falling below 80% of their daily recommended intake for macronutrients, as well as increase the risk of micronutrient deficiencies essential to growth and bone development, including vitamins A, C, D, K, B12, zinc, iron, calcium, and potassium [9–11]. These nutritional shortfalls are of particular concern in ARFID given the early age of onset and the chronicity of highly restrictive eating patterns. Consequently, biological processes dependent upon nutrition adequacy could be impacted.

The childhood and adolescent years are a pivotal stage of musculoskeletal development characterized by dynamic changes in bone elongation and mineralization [12, 13]. Growth in stature and bone mass accrual are processes that are highly dependent on nutrition adequacy [12]. Peak height velocity, or the age at which height accrual is most rapid, is achieved around the early teenage years and is closely related to puberty and bone mass accrual [13]. Approximately 35% of adult bone mass is gained during the four years surrounding peak height velocity [13]. Bone mass tends to increase non-linearly during the growing years, with greater than 95% of adult bone mineral density (BMD) accrued by the age of 20, at which point “peak bone mass” is attained [12, 13]. Since bone density is generally stable or “tracks” across the lifespan, peak bone mass is a determinant of fracture and osteoporosis risk in adulthood [14–16]. Accordingly, nutrition-related threats to peak bone mass attainment can lead to musculoskeletal consequences across the lifespan.

**Fig. 1:**
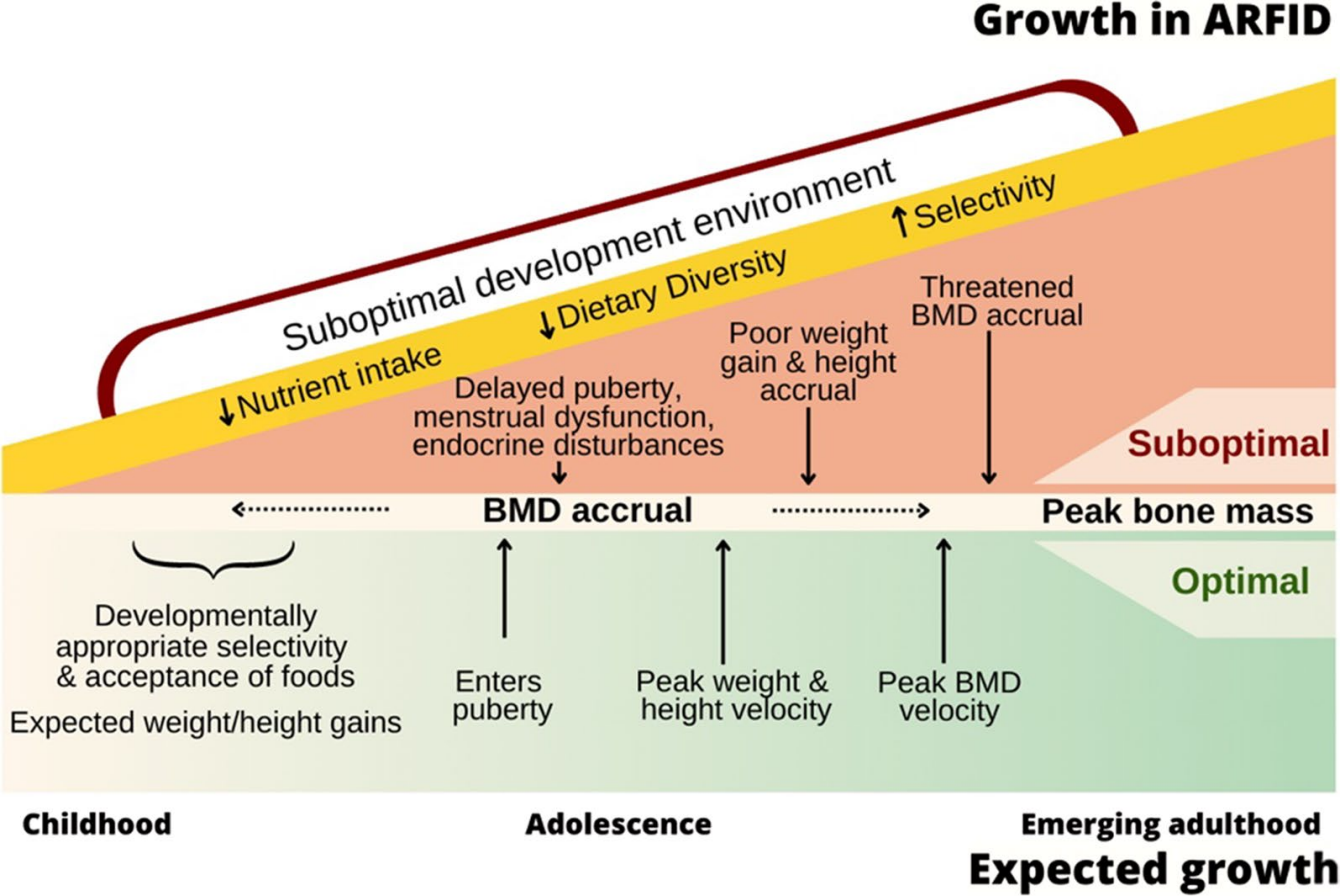
Schematic showing potential ARFID-related factors threatening peak bone mass attainment

The introduction of ARFID into the diagnostic nomenclature and robust evidence implicating other forms of feeding/eating disorders as threats to bone health [17] collectively highlight the need to understand effects of ARFID on bone health. This narrative review summarizes available clinical evidence involving ARFID and related nutritional deficiencies and bone health outcomes (Fig. 1), and suggests important “next steps” in this line of study. Articles were identified by searching Pubmed, and to the best of our knowledge, only two studies reported BMD outcomes in individuals with ARFID.

## Bone health assessment methodology

Osteoporosis is commonly referred to as the “silent disease.” Low bone density and impaired bone quality often go unnoticed due to lack of physical signs and symptoms. Readily accessible demographic (e.g., race, sex, age), clinical (e.g., medications, health status), and physical (e.g., height, weight, BMI) characteristics could help inform a clinician on a patient’s fracture risk, but imaging-based methods are required for BMD monitoring and osteoporosis diagnosis. Dual-energy X-ray absorptiometry (DXA) is the gold standard clinical and research tool used to assess BMD in pediatric and adult patients. The ability of DXA to assess BMD at various anatomic regions is a strength of this technique since the composition of the skeleton with respect to cortical and trabecular bone varies across regions. In pediatric patients, the International Society for Clinical Densitometry (ISCD) recommends assessing the total body (excluding the head) and the lumbar spine, which are skeletal regions primarily comprised of cortical and trabecular bone, respectively [18]. Due to the close relationship between BMD and stature, the ISCD also recommends adjusting BMD measures for height to minimize potential confounding, particularly in children and adolescents with pubertal delay or short stature. This includes computing height Z-score-adjusted BMD Z-scores using published calculations [19], as well as calculating lumbar spine bone mineral apparent density (BMAD). Pediatric growth charts for lumbar spine BMAD for American children were recently published. BMAD demonstrates strong tracking across childhood and adolescence, similar to other BMD measures, while helping minimize stature-related artifacts in BMD [14].

Robust reference ranges help account for variability in BMD during the dynamic growing years. Similar to growth references from the Centers for Disease Control and Prevention and growth standards from the World Health Organization, BMD growth charts help account for the non-linear increases in bone density that occur in childhood, in addition to sex and race differences. Standard deviation scores, or Z-scores, are calculated from reference datasets and facilitate the interpretation of measurements such as BMD. A Z-score indicates the number of standard deviations above or below the age-specific mean that a BMD value sits on the growth chart. BMD growth charts are typically specific for sex and race, with some exceptions. Based on a standard normal distribution, it is expected that nearly 96% of people from the generally healthy population will be within ± 2 standard deviations of the mean, and that only about 2% will be greater than 2 standard deviations less than (or above) the mean. Thus, a Z-score of 0 represents the 50th percentile, and Z-scores of − 2 and + 2 are approximately equivalent to the 3rd and 97th percentiles, respectively. Additional strengths of DXA include wide-spread availability, low cost, marginal radiation dose, fast scan times, and the assessment of lean and fat mass. Understanding these assessment methodologies is important to contextualize findings currently reported in the literature related to bone health in people with ARFID. Consideration of available technologies also informs the development of recommendations for clinical use.

## The impact of eating/feeding disorders on bone health and growth

As defined by the DSM-5, restrictive eating disorders include AN-Restrictive (AN-R), AN-Atypical (AN-A), and ARFID [20]. AN-R and AN-A are characterized by intentional energy restriction and fear of weight gain, with AN-R associated with more severe low body weight. Concerning ARFID, “faltering growth” is noted as one of four potential diagnostic manifestations and is defined as “significant weight loss,” “failure to achieve expected weight gain,” or “faltering growth in children” [1]. There is considerable variability in body size within the ARFID population, as poor growth is not required to meet diagnostic criteria for ARFID.

Longitudinal growth depends upon adequate nutrition, therefore height serves as an indicator of healthy development and nutritional status. In addition to its association with shorter adult stature, growth stunting during the formative years is associated with many adverse outcomes, including disturbances in puberty and sexual maturation, cognitive deficits, and poor academic performance. Many key nutrients and food groups involved in longitudinal growth are not consumed in adequate amounts in people with severe food selectivity such as in ARFID. This includes, but is not limited to, protein, zinc, iodine, calcium, vitamin D, and dairy [9, 21, 22]. Although no studies thus far have explicitly focused on longitudinal growth in children and adolescents with ARFID, several studies have reported descriptive statistics relating to stature, suggesting that children and adolescents with ARFID have shorter stature than healthy reference datasets. A recent study by Alberts and colleagues compared various growth measures between children and adolescents from the United Kingdom ages 6–19 years that were referred to a clinical specialist due to health concerns resulting from body weight and disordered eating/feeding [23]. In this study, AN (n = 118) and ARFID (n = 16) classifications included components of “decreased body weight” and “clinically significant underweight,” respectively [23]. Average BMI Z-score, calculated using United Kingdom growth reference data, was greater than 1.5 standard deviations below the population mean for both groups. Although the number of patients with a height Z-score less than − 2.0 was not reported, the average height of children with ARFID was nearly one standard deviation below the healthy population, similar for age and sex, with average eight Z-score being − 0.88 ± 1.15 in the ARFID group. Height Z-scores in the ARFID group were slightly lower than the AN group, but did not differ significantly. Interestingly, stature deficits in the ARFID group were more pronounced in males compared to females. This is an important finding since boys are more prone to fracture compared to girls [13], and ARFID is observed at higher rates in males than in females, in contrast to other eating disorders.

Dinkler and colleagues used a subset of data from the Japan Environment and Children’s Study, a nationwide birth cohort study, to describe the prevalence and characteristics of ARFID in 6,633 children ages 4–7 years [24]. In this study, children with ARFID tended to be shorter than those without ARFID, and differences in height between AFRID subtypes were evident. Individuals classified as having either A1, A2, or A3 subtypes, which include components of low body weight, nutritional deficiencies, and/or reliance on enteral or oral nutrition supplementation, tended to have shorter stature compared to those with the A4 subtype, which is based on the presence of impairment in psychosocial functioning due to disturbances in eating/feeding without documented nutritional compromise or formula supplementation. These differences support the notion that ARFID has varying effects on growth in stature depending upon its subtype, with the most pronounced deficits evident in those diagnosed with ARFID subtypes A1–A3.

Studies involving bone health in individuals with eating disorders have primarily focused on people with AN, revealing relatively consistent bone deficits in these patients [17, 25, 26]. Individuals with AN-R and AN-A have bone deficits when compared to non-AN healthy controls, with AN-R typically having lower BMD compared to those with AN-A [27]. The lower BMD in both AN groups suggests that body weight alone does not fully account for threats to bone health and underscores the importance of studying the effects of all types of restrictive intake on bone health. In one of the first studies of bone outcomes in people with ARFID, Schorr and colleagues conducted a cross-sectional study of adults ages 18–63 years with clinically diagnosed eating disorders from two hospitals in the United States [27]. Compared to healthy controls matched for race and age (n = 48), men with AN-R (n = 26) and ARFID (n = 11), but not AN-A (n = 18), had lower hip and spine BMD Z-scores. Average spine BMD Z-scores for the AN-R and ARFID groups were − 2.05 ± 1.58 and − 1.33 ± 1.21, respectively, and a greater proportion of AN-R and ARFID men had BMD Z-scores less than − 2.0 compared to the healthy control group. It is important to note that the specific ARFID subtype of these patients was not stated in the article. Classification of ARFID was based on the presence of restricted eating without psychological symptoms consistent with AN. There were no body weight criteria for ARFID classification, but the ARFID group had a similar BMI to the AN-R group and a lower BMI than the AN-A group. Since ARFID typically manifests in childhood, the lack of specificity with regards to ARFID manifestation and the older age of these patients complicates the translation of these findings.

The study by Alberts and colleagues discussed above expanded upon the results of Schorr et al. [23] by comparing lumbar spine bone density measures between youth with ARFID and AN. In the main analyses, bone Z-scores were generally low in both groups, but did not differ significantly. To account for potential confounding of key variables, these authors conducted secondary analyses matching one patient with ARFID (n = 13) to one patient with AN (n = 13) based on age, sex, and pubertal stage and compared bone Z-scores across groups. In the ARFID and AN group, the lumbar spine BMD Z-score was − 1.74 ± 1.04 and − 1.40 ± 1.21 (*P* = 0.22), respectively, and the lumbar spine BMAD Z-score was − 1.44 ± 0.86 and − 1.03 ± 1.53 (*P* = 0.07), respectively. Overall, bone Z-scores tended to be lower in the ARFID versus the AN group, but these differences were not statistically significant. Accordingly, these findings suggest that bone deficits in youth with ARFID are of a similar magnitude as those observed in youth with AN. In addition, because bone deficits were evident when considering lumbar spine BMAD, which helps account for short stature and pubertal delay, this suggests that threatened BMD in children with ARFID is likely independent of stature-related artifacts. A summary of these studies is presented in Table 1.

**Table 1:** Summary of studies reporting bone health outcomes in people with ARFID

Authors	Study Design	Study Description	Sample Description	Sample Size	Endpoints		Results	
						HC	AN	ARFID
Schorr et al. [27]	Cross-sectional	This study investigated the prevalence of low BMD, and its determinants, in men with AN, atypical AN, and ARFID	Sex: 100% Male Age: 18–63 Race: 100% White Location: Massachusetts General Hospital (Boston, MA) and Denver Health Medical Center (Denver, CO)	HC: N = 48 AN: N = 26 ARFID: N = 11	Prevalence of PA spine BMD Z-score less than − 1 and − 2	< − 1: 23% < − 2: 6%	< − 1: 77%^a^ < − 2: 62%^a^	< − 1: 64% < − 2: 18%
					Prevalence of total hip BMD Z-score less than − 1 and − 2	< − 1: 8% < − 2: 2%	< − 1: 50%^a^ < − 2: 15%	< − 1: 64%^a^ < − 2: 9%
					Prevalence of femoral neck BMD Z-score less than − 1 and − 2	< − 1: 10% < − 2: 2%	< − 1: 58%^a^ < − 2: 23%^a^	< − 1: 45%^a^ < − 2: 0%
Alberts et al. [23]	Cross-sectional retrospective case-note review	This study compared BMD between patients with ARFID vs. AN	Sex: 19% Male Age: 6–19 Race: 87% White Location: Great Ormond Street Hospital (London, Eng.)	AN: N = 118 ARFID: N = 16			AN	ARFID
					Spine (L2-L4) BMD (mean, SD)		0.92^b^, 0.17	0.70, 0.13
					Spine (L2-L4) BMD Z-score (mean, SD)		− 1.43, 1.18	-1.88, 0.91
					Spine (L2-L4) BMAD Z-score (mean, SD)		− 1.03, 1.53	-1.44, 0.88

^a^Differed significantly from healthy controls at *P* < 0.05

^b^Differed significantly from ARFID group at *P* < 0.05

*AN* Anorexia nervosa, *ARFID* Avoidant/restrictive food intake disorder, *BMD* Bone mineral density, *BMAD* Bone mineral apparent density, *DXA* Dual-energy X-ray absorptiometry, *HC* Healthy control, *PA* Posterior-anterior, *SD* Standard deviation

## Mediators of bone deficits in ARFID

### Nutritional status

The skeleton is responsive to static and dynamic loading from standing, locomotion, physical activity, and muscle contractions [12, 28]. Although the body size phenotype in ARFID is highly variable, all individuals with the ARFID A1 subtype have low body weight and/or BMI. In studies of patients with AN, current body weight and a history of low body weight are among the strongest determinants of BMD [17, 29]. Similar findings have been reported in people with ARFID. Alberts and colleagues report strong associations between BMI, BMI Z-score, and underweight duration as predictors of BMD Z-score, and BMI and BMI Z-score as predictors of BMAD Z-score [23]. Similarly, in Schorr et al. [27], current BMI and past BMI were strongly associated with bone outcomes at the femoral neck, hip, and spine in adults. In addition, these authors report lower lean mass and percent body fat in adults with ARFID compared to controls and found significant positive correlations between appendicular lean mass and bone Z-score. These studies highlight the role of body weight in ARFID-related bone deficits and suggest skeletal muscle deficits might be involved in these relationships. Measures of body composition should be considered in future studies since fat mass and lean mass play a prominent role in peak bone mass attainment and maintenance.

Vitamin C deficiency is among the most common nutrient deficiencies in patients with ARFID in the form of severe food selectivity [30]. The skeletal matrix comprises an integrated framework of type 1 collagen, which serves as scaffolding for the mineral hydroxyapatite component of bone. Vitamin C is involved in collagen synthesis and modification of bone turnover through the wnt/β-catenin signaling pathway [31, 32]. Although vitamin C deficiency is uncommon in the general population, several recent clinical case reports discuss the development of scurvy in patients with ARFID and its subsequent effects on bone health [30, 33]. Scurvy is associated with numerous musculoskeletal manifestations evident through radiographic testing and patient observation, such as joint swelling and bone pain. What was once considered a historical diagnosis, vitamin C deficiency should be considered in patients with ARFID who are restricting and/or omitting foods that are rich in vitamin C, such as fruits, vegetables, and juices [30, 33].

Based on the pattern of food restriction, another historically associated vitamin deficiency that can occur in patients with ARFID is vitamin D deficiency rickets. Vitamin D is a pro-hormone nutrient involved in modulating bone turnover and augmenting intestinal calcium absorption. Cutaneous conversion of 7-dehydrocholesterol to cholecalciferol via UVB radiation is the primary source of vitamin D for humans, but dietary sources include fatty fish, eggs, soy, dairy, and juices [34]. Under-mineralized bone in children with vitamin D deficiency may lead to the development of rickets [35]. Children with rickets exhibit bowing of the legs, curving of the spine, and deficits of dentition [36]. Vitamin D deficiency in childhood can also cause growth restriction and skeletal deformities, increasing the risk of fracture [35, 37]. In the previously discussed study by Schorr and colleagues, serum vitamin D was measured alongside BMD in men with AN and ARFID [27]. In men with AN, serum vitamin D less than 20 ng/mL were common, along with deficits in BMD and estimated bone bending strength, even after accounting for BMI [27]. Recently, associations between low vitamin D status, allergies, and autoimmune disorders have been recognized in children [37]. Allergies and autoimmune dysfunction are common comorbidities in children with ARFID [38]. Although vitamin D status in individuals with ARFID has not been extensively studied, several published clinical cases report vitamin D deficiency rickets in children and adolescents with autism spectrum disorder (ASD) with severe food selectivity. It should be noted that ASD is a common ARFID comorbidity [38, 39].

### Endocrine

Endocrine disruptions are common in patients with eating disorders due to low energy availability [40]. Suboptimal production and/or action of growth hormone, insulin-like growth factor 1 (IGF-1), IGF-1 related binding proteins, parathyroid hormone, cortisol, testosterone, estrogen, and gonadotropin-releasing hormone can contribute to, or manifest in, pubertal delay and growth disturbances [41]. A recent study by Katzman and colleagues reported greater than 50% of girls with ARFID, who had already reached menarche, had secondary amenorrhea [42]. In a separate study, Aulinas and collogues compared endocrine dysfunction in females ages 10–22 years with ARFID (n = 20), AN (n = 42), and healthy controls (n = 49) without a history of disordered eating [43]. Compared to females with AN and controls, females with ARFID had fewer missed menses in the preceding nine months, higher triiodothyronine (T3), and a lower total thyroxine (T4) to T3 ratio, which could reflect delayed conversion of thyroid hormone to its active form [43]. These endocrine disruptions during puberty could contribute to faltering longitudinal growth and bone accrual in individuals with ARFID.

### Feeding issues in complex medical conditions

Feeding disruption is estimated to occur in between 40 and 80% of children with complex medical and/or developmental conditions [44]. This pattern of comorbidity has been observed in pediatric feeding disorder treatment settings and adolescent medicine eating disorder programs [44, 45]. Common medical comorbidities associated with feeding disorders include neurodevelopmental disorders (e.g., autism spectrum disorder, cerebral palsy, intellectual disability), prematurity, cardiopulmonary disease, food allergies (e.g., IgE mediated and non-IgE mediated such as eosinophilic esophagitis, celiac disease, food protein-induced enterocolitis), and gastroenterological conditions (e.g., reflux and vomiting) [44, 45]. Many of these comorbid conditions have been noted to increase the risk for bone deficits, including ASD, prematurity, food allergies, and inflammatory bowel diseases [46–49]. However, it is not well understood how restrictive intake patterns such as those observed in patients with ARFID may exacerbate and interact with risks conferred by disease status. It is also worth noting that certain medications required to manage a patient’s medical condition may also impact food intake and growth via effects on hunger/satiety, nutrient metabolism and absorption, and mealtime restrictions. For example, children with attention-deficit/hyperactivity disorder and ARFID may be prescribed stimulant medications to manage hyperactive/inattentive symptoms, which commonly suppress appetite and may impact overall energy intake. Several studies implicate the use of stimulants in suboptimal bone health during the years preceding peak bone mass [50–52].

## Clinical considerations

Multidisciplinary interventions consisting of psychologists, dietitians, physicians, speech-language pathologists, and occupational therapists provide a comprehensive treatment framework and represent the gold standard of care for pediatric feeding disorders, including ARFID [44]. Behavioral, cognitive-behavioral, and family systems-based interventions delivered in outpatient, intensive outpatient, and inpatient care settings across pediatric and adolescent medicine patient populations show particular promise in facilitating nutritional stabilization and the development of appropriate feeding practices [44, 53–56]. Improvements in feeding behaviors will help address accompanying health concerns of low body weight, nutritional deficiencies, and/or reliance on nutritional supplements. Of note, findings in the AN literature have demonstrated that regaining body weight is beneficial to bone health outcomes, but bone deficits are not likely fully restored [29]. The effects of feeding intervention on longitudinal growth and bone accrual in youth with ARFID have yet to been studied.

As discussed above, DXA is the preferred method of bone health assessment in pediatric and adult patients, but there are currently no published guidelines for bone health assessment and management of patients with ARFID. For patients with eating disorders, it is generally recommended that a DXA evaluation for bone health assessment should be considered if illness duration exceeds one year or if amenorrhea is present for greater than six months [57]. From a broader standpoint, the ISCD guidelines indicate that for all children and adolescents with a chronic disease that is suspected to impact bone biology, bone health assessment via DXA should be performed when a patient might benefit from intervention to minimize fracture risk, and when results from a DXA evaluation will help guide clinical management [18]. Since youth with ARFID are more prone to having shorter stature, consideration of height-adjusted BMD measures is important to minimize stature-related confounding.

Although ISCD guidelines recommend DXA assessments of the total body (less head) and lumbar spine for pediatrics and the lumbar spine and hip for adults, other skeletal regions such as the forearm could provide valuable complementary information with respect to bone health. In pediatric patients, measurements of radius BMD can be valuable since fractures are most often sustained at the forearm during the vulnerable period of the childhood growth spurt [13]. The forearm is not habitually exposed to static and/or dynamic impact loading, which might be an important consideration for patients who have recently experienced a significant weight loss (or gain) since BMI is a main determinant of BMD in people with eating/feeding disorders [23, 27]. The forearm is also unique in that the mid-region (the diaphysis) of the radius is predominantly comprised of cortical bone, but the ends (the metaphysis) is mainly trabecular bone. Cortical and trabecular bone can be differently impacted by disease pathophysiology, clinical intervention, and environmental and/or behavioral factors, underscoring the potential utility of forearm BMD measures in complex conditions of multifactorial pathophysiology. Robust reference datasets for pediatric forearm DXA scans are now readily available [16, 58].

**Fig. 2:**
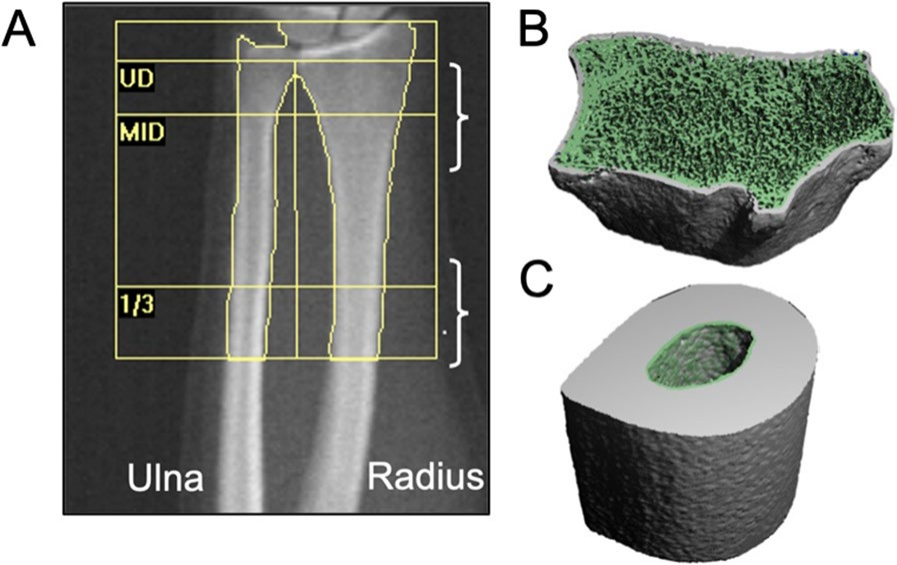
Representative forearm DXA (**A**) and HR-pQCT (**B**, **C**) images are displayed on the left and right, respectively. In the DXA image, the radius and ulna are labeled accordingly, and brackets identify the approximate regions of the radius at which HR-pQCT scans were performed. HR-pQCT scans were performed at 3.5% (**B**) and 30% (**C**) regions of the radius (based on forearm length and relative to the distal end plate), for assessment of trabecular and cortical bone, respectively. Presented DXA and HR-pQCT images were from an otherwise healthy 23-year-old female that had a BMI of 23 kg/m^2^. All scans were performed at the University of Georgia’s Nutrition and Skeletal Health Laboratory by a trained graduate research assistant using a Hologic Horizon DXA and Scanco XTremeCT-II HR-pQCT densitometers

Despite the many strengths of DXA, it is not without limitations. The skeleton is a three-dimensional object, yet DXA only provides a two-dimensional depiction of the bone. Thus, the “bone density” metric derived from DXA is an “areal” measure of density rather than a volumetric measure of density and is therefore measured in units of grams per centimeters squared (g/cm^2^). This is the main limitation of DXA that is addressed in the research setting through the use of alternate bone imaging modalities. The high-resolution peripheral quantitative computed tomography (HR-pQCT) is a relatively new technology that assesses bone micro-structural features of the skeleton that are otherwise unattainable from standard DXA techniques. Regions of the appendicular skeleton, including the distal radius and distal tibia, are often evaluated using HR-pQCT to assess macro and micro-structural features of the cortical and trabecular bone. Based on these morphologic and densitometric characteristics, finite element analysis algorithms are utilized to estimate bone strength. These measures are closely related to fracture and provide information on bone fragility beyond standard BMD metrics [59]. Figure 2 presents sample DXA and HR-pQCT images acquired at the distal forearm. To this point, HR-pQCT methods have not yet been applied in studies involving children or adults with ARFID. Advancements in the field to better understand the implications of ARFID on health, including attainment and maintenance of peak bone mass and peak bone strength through application of contemporary DXA and HR-pQCT methods, would enable development of guidelines for the medical management of individuals with ARFID similar to existing guidelines for AN.

Bone imaging is not currently the standard of care for individuals with ARFID, but could improve early identification of the health impacts of selective eating and enable individuals to access care sooner rather than waiting until more severe, later onset problems resulting from under-nutrition (e.g., fracture) emerge. Additionally, bone imaging as part of the initial health assessment of individuals with highly restrictive eating, particularly if restriction has been long-standing, could help tailor and prioritize intervention approaches. Since low body weight (or, BMI) and short stature are associated with lower BMD, these readily available and inexpensive anthropometric measurements may help identify patients that are at greatest risk in the clinical setting, and therefore necessitate a DXA exam.

## Conclusions

Since the introduction of ARFID into the diagnostic nomenclature, considerable progress has been made in describing the short-term health consequences of malnutrition. The rich body of clinical evidence highlighting bone health deficits in people with AN underscores the need for focused research involving skeletal development in individuals with ARFID [29]. Early evidence suggests that children and adults with ARFID have lower bone density and shorter stature compared to healthy counterparts and that bone deficits in people with ARFID are similar in magnitude to those observed in patients with AN. Although these limited studies provide an essential foundation of evidence, many critical knowledge gaps and questions remain. To what extent does ARFID's effect on bone health depend upon the specific subtype? Are BMD deficits in children with ARFID persistent across the growing years and associated with risk for osteoporosis and fracture in adulthood? What characteristics of cortical and/or trabecular bone morphology are impacted in individuals with ARFID? What are the underlying biological and behavioral mechanisms involving growth deficits in children with ARFID? Do improvements in feeding and nutritional status resulting from clinical intervention restore bone deficits? To address these and other research questions, prospective studies in children, adolescents, and adults with ARFID are required to understand the natural course of progression and the role of clinical intervention in minimizing threats to bone health. These efforts will help inform comprehensive medical assessment, improve long-term health outcomes, and provide a benchmark for assessing treatment outcomes over time.

## Data Availability

Not applicable.

## Abbreviations

ARFID: Avoidant/restrictive food intake disorder
AN: Anorexia nervosa
AN-R: Anorexia nervosa restrictive
AN-A: Anorexia nervosa atypical
DSM: Diagnostic and statistical manual
DXA: Dual-energy X-ray absorptiometry
BMD: Bone mineral density
ISCD: International society for clinical densitometry
BMAD: Bone mineral apparent density
HR-pQCT: High resolution peripheral quantitative computed tomography
ASD: Autism spectrum disorder
IGF-1: Insulin-like growth factor 1
